# Vitamin D and circulating tumor cells in primary breast cancer

**DOI:** 10.3389/fonc.2022.950451

**Published:** 2022-09-07

**Authors:** Michal Mego, Barbora Vlkova, Gabriel Minarik, Zuzana Cierna, Marian Karaba, Juraj Benca, Tatiana Sedlackova, Dana Cholujova, Paulina Gronesova, Katarina Kalavska, Daniel Pindak, Jozef Mardiak, Peter Celec

**Affiliations:** ^1^2^nd^ Department of Oncology, Faculty of Medicine, Comenius University and National Cancer Institute, Bratislava, Slovakia; ^2^ Translational Research Unit, Faculty of Medicine, Comenius University and National Cancer Institute, Bratislava, Slovakia; ^3^ Institute of Molecular Biomedicine, Faculty of Medicine, Comenius University, Bratislava, Slovakia; ^4^ Department of Pathology, Faculty of Medicine, Comenius University, Bratislava, Slovakia; ^5^ Department of Pathology, Faculty Hospital, Trnava, Slovakia; ^6^ Department of Oncosurgery, National Cancer Institute, Bratislava, Slovakia; ^7^ Department of Medicine, St. Elizabeth University, Bratislava, Slovakia; ^8^ Cancer Research Institute, Biomedical Research Center of the Slovak Academy of Sciences, Bratislava, Slovakia; ^9^ Department of Oncosurgery, Slovak Medical University, Bratislava, Slovakia

**Keywords:** primary breast cancer, vitamin D, circulating tumor cells, prognosis, epithelial-mesenchymal transition

## Abstract

**Background:**

Circulating tumor cells (CTCs) contribute to the metastatic cascade and represent an independent survival predictor in breast cancer (BC) patients. Vitamin D has pleiotropic effects, and its low concentrations are associated with breast cancer and metastasis. The aim of this study was to assess plasma vitamin D in primary BC patients in relation to CTCs.

**Methods:**

This study included 91 non-metastatic BC patients (stage I–III) and 24 healthy donors. Blood samples for the analyses were drawn at the time of surgery. CTCs were assessed using a quantitative RT-PCR assay for expression of epithelial (*CK19*) or epithelial-to-mesenchymal transition (EMT) genes (*TWIST1*, *SNAIL1*, *SLUG*, and *ZEB1*). Total 25-OH vitamin D was measured in plasma using ELISA. Plasma cytokines and angiogenic factors were measured by enzyme-linked immunoassay.

**Results:**

CTCs were detected in 30 (33%) patients. Patients with detectable CTCs in peripheral blood had significantly lower vitamin D concentrations in comparison to patients without detectable CTCs ((mean ± SD) 8.50 ± 3.89 µg/L for CTC-positive vs 9.69 ± 3.49 µg/L for CTC-negative patients, p = 0.03). The mean ( ± SD) vitamin D plasma level was 9.3 ± 3.65 µg/L for breast cancer patients compared to 18.6 ± 6.8 for healthy donors (p < 0.000001). There was no association between plasma vitamin D and other patient/tumor characteristics. Plasma vitamin D levels are inversely correlated with plasma TGF-β1, TGF-β2, IL β, IL-5, and eotaxin (all p < 0.05). Patients with vitamin D above the median had a better overall survival (hazard ratio (HR) = 0.36, 95% CI 0.16–0.80, p = 0.017), and combined analysis showed the best survival for CTC-negative patients with vitamin D levels above the median as compared to patients with opposite characteristics (HR = 0.18, 95% CI 0.05–0.63, p = 0.004).

**Conclusions:**

Low vitamin D could be a consequence and hence a biomarker of a more invasive disease. Alternatively, vitamin D could be associated with survival because of its role in tumor dissemination. Whether its supplementation affects the metastatic cascade should be tested in animal experiments and interventional studies.

## Introduction

Breast cancer is one of the most common cancers and the leading cause of cancer death among women in developed countries (1). Metastatic disease is responsible for morbidity and mortality, and despite advances in treatment in the last decades, it remains an incurable condition for the vast majority of metastatic breast cancer patients (1).

Circulating tumor cells (CTCs) contribute to the metastatic cascade and represent an independent survival predictor in primary and metastatic breast cancer patients (2–7). CTCs represent a heterogeneous population of cancer cells with different biological and clinical values (8). The majority of current detection methods are able to identify CTC with epithelial phenotype (3, 6). However, due to epithelial-to-mesenchymal transition (EMT), a subpopulation of CTC could suppress their epithelial characteristics and gain mesenchymal features and cancer stem cell phenotype (9–13).

Vitamin D has pleiotropic effects, and its low concentrations are associated with infections, cancer, inflammation, and other pathologic conditions (14–16). There are numerous studies that showed an inverse relationship between vitamin D levels and inflammation, autoimmune disease, and cancer (17–22). Vitamin D signaling could be divided into non-genomic and genomic (23).

Epidemiological studies suggest an inverse association between breast cancer incidence and vitamin D levels (24). Similarly, a low level of vitamin D was associated with inferior outcomes in breast cancer (25, 26). While the correlation between vitamin D levels and breast cancer is established, currently, data showing that vitamin D supplementation is associated with improved outcomes in primary or metastatic breast cancer are lacking (19).

Preclinical studies suggest that vitamin D has a suppressive effect on several aspects of metastatic cascade (27, 28). This is achieved through suppression of matrix metalloproteinases (MMPs), urokinase plasminogen activator (uPA) system, inhibitory effect on EMT, and differentiation effect on cancer stem cells (29, 30). However, we lack data on the association between CTC and vitamin D levels. The aim of this study was to assess plasma vitamin D levels in primary breast cancer (BC) patients in relation to CTCs. We also aimed to determine the relationship between plasma vitamin D and selected tumor matrix metalloproteinases, uPA system, and plasma cytokines, as all of these factors could be affected by vitamin D.

## Methods

### Study patients

This study included 91 non-metastatic breast cancer patients (stage I–III) who underwent surgery from March to November 2012 and for whom plasma isolated on the day before surgery was available in the biobank. This study is a part of a larger translational study (Protocol TRU-SK 002; Study chair: M. Mego, date of approval 20 June 2011) and aimed to determine the prognostic value of circulating tumor cells in primary breast cancer as described previously (11). This substudy included 24 healthy donors for whom plasma was available in the biobank. The study was approved by the Institutional Review Board (IRB) of the National Cancer Institute of Slovakia. Each participant provided signed informed consent before study enrollment.

### Circulating tumor cell detection in peripheral blood

The presence of CTCs in peripheral blood was determined by a quantitative real-time polymerase chain reaction (qRT-PCR)-based assay as described previously (11, 31, 32). The highest expression values in healthy donors were used as “cutoff” to determine CTC positivity. Patient samples with *CK19* gene transcripts higher than those of healthy donors were scored as epithelial CTC (CTC_EP)-positive, while patient samples with higher EMT gene transcripts than those of healthy donors were scored as CTC_EMT-positive.

The highest expression levels of the KRT19- and EMT-inducing TF gene transcripts relative to that of GAPDH were 3.4 × 10^−3^ (median 2.8 × 10^−6^, range 0–3.4 × 10^−3^) for KRT19, 7.5 × 10^−4^ (median 0, range 0–7.5 × 10^−4^) for TWIST1, 3.8 × 10^−2^ (median 3.1 × 10^−3^, range 5.0 × 10^−4^–3.8 × 10^−2^) for SNAIL1, and 1.7 × 10^−1^ (median 1.4 × 10^−2^, range 2.2 × 10^−3^–1.7 × 10^−1^) for ZEB1, while SLUG transcripts were not detected in any of the samples from a healthy donor.

### Plasma isolation

Peripheral venous blood samples were collected in EDTA-treated tubes in the morning on the day of surgery, centrifuged at 1,000 *g* for 10 min at room temperature within 2 h of venipuncture, and processed as described previously (11, 31, 32).

### Vitamin D plasma level measurement

Total 25-OH vitamin D was measured in plasma using ELISA (Demeditec Diagnostics, Kiel, Germany). The intraassay and interassay coefficients of variation were below 3% and 10%, respectively. According to the World Health Organization, levels <10 and <20 ng/ml are considered deficient and insufficient, respectively (33).

### Measurement of serum calcium

Serum calcium was measured in a standard hospital biochemistry laboratory by Atellica™ CH Calcium (Ca) test (Siemens, Munich, Germany), which is based on spectrophotometry. Serum calcium was corrected to albumin level by the following formula: corrected calcium mmol/L = (0.02 * (normal albumin − patients albumin)) + serum calcium (34).

### Measurement of urokinase plasminogen activator, plasminogen activator inhibitor-1, and plasma cytokines and angiogenic factors

Plasma uPA and plasminogen activator inhibitor-1 (PAI-1) were analyzed using enzyme-linked immunosorbent assays (ELISA) as described previously (35). Briefly, plasma tissue factor (TF) was analyzed by ELISA using the Quantikine Human Coagulation Factor III/Tissue Factor Immunoassay (R&D Systems, Minneapolis, MN, USA). D-dimer in plasma samples was determined using the IMUCLONE D-Dimer ELISA (American Diagnostica, Greenwich, CT, USA) system. Plasma uPA was measured using the Human u-Plasminogen Activator/Urokinase Quantikine ELISA kit (R&D Systems). Plasma PAI-1 was measured using the Human Serpin E1/PAI-1 Quantikine ELISA Kit (R&D Systems).

Plasma samples were analyzed for 51 plasma cytokines and angiogenic factors (TGF-β1, TGF-β2, TGF-β3, IFN-α2, IL-1α, IL-2Rα, IL-3, IL-12p40, IL-16, IL-18, CTACK, Gro-α, HGF, LIF, MCP-3, M-CSF, MIF, MIG, β-NGF, SCF, SCGF-β, SDF-1α, TNF-β, TRAIL, IL-1β, Il-1RA, IL-2, IL-4, IL-5,IL-6, IL-7, IL-8, IL-9, IL-10, IL-12, IL-13, IL-15, IL-17, eotaxin, FGF basic, G-CSF, GM-CSF, IFN-γ, IP-10, MCP-1, MIP-1α, MIP-1β, PDGF bb, RANTES, TNF-α, and VEGF) using pre-designed panels as described previously and were available for subset of patients (Bio-Plex Pro TGF-β assay, Bio-Plex Pro Human Cytokine 21- and 27-plex immunoassays; Bio-Rad Laboratories, Hercules, CA, USA) (35).

### Tissue MMP1 and MMP9 evaluation

A pathology review was conducted at the Department of Pathology, Faculty of Medicine, Comenius University, by a pathologist associated with the study. The study included tumor specimens corresponding to plasma samples from 78 patients. All specimens were classified according to the WHO Classification of 2004. The block containing the most representative part of the hematoxylin and eosin (H&E)-stained tumor was identified by microscopy and subsequently used for immunohistochemistry (IHC) analysis. Tissue microarray construction and immunohistochemical staining were performed as described previously (31, 36). MMP1 was detected by primary rabbit polyclonal antibody against MMP1 (LSBio, MMP1, LS-B1229) diluted 1:40 in Dako REAL antibody diluent (Dako, Glostrup, Denmark), while MMP9 was detected by primary mouse monoclonal antibody against MMP9 (Abcam; MMP9 (SB15c); cat. no. ab51203) diluted 1:200 in Dako REAL antibody diluent (Dako; Agilent Technologies, Inc., Santa Clara, CA, USA) as described previously (31, 36).

The result of the immunohistochemical analyses was expressed by a weighted histoscore, evaluating both the percentage of positive cells and the staining intensity of the nuclei or cytoplasm as described previously (31, 36).

### Statistical analysis

The characteristics of patients were summarized using mean (range) for continuous variables and frequency (percentage) for categorical variables.

The normality of data distribution was determined by the Kolmogorov–Smirnov test. Data demonstrating normal distribution were analyzed by Student’s t-test or analysis of variance, while non-normally distributed parameters were statistically evaluated by the non-parametric Mann–Whitney U-test or Kruskal–Wallis H test. Pearson’s or Spearman’s correlations tests were used according to the normality of data.

The median follow-up period was calculated as the median observation time among all patients and among those who were still alive at the time of their last follow-up. Disease-free survival (DFS) was calculated from the date of blood sampling to the date of disease recurrence (locoregional or distant), secondary cancer, death, or last follow-up. Overall survival (OS) was calculated from the date of blood sampling to the date of death or last follow-up. DFS and OS were estimated using the Kaplan–Meier product limit method and compared between groups by log-rank test.

A multivariate Cox proportional hazards model for DFS and OS was used to assess differences in the outcome on the basis of the vitamin D status (“high” defined as above median vs. “low” below median), hormone receptor status (positive for either vs. negative for both), HER2 status (positive or negative), axillary lymph node involvement (N0 vs. N+), and grade (Grade 3 vs. grade 1 and 2). All p-values presented are two-sided, and associations were considered significant if the p-value was less than or equal to 0.05. Statistical analyses were performed using NCSS 11 Statistical Software (2016, NCSS, LLC, Kaysville, UT, USA; ncss.com/software/ncss).

## Results

### Patient characteristics

The study population consisted of 91 primary breast cancer patients: median age was 60 years (range 25–83 years), while the median age of healthy donors was 54 years (range 25–66 years, p = 0.004). **Table 1** summarizes patient characteristics. The majority of patients had T1, node-negative, and hormone receptor-positive primary tumors. CTCs were detected in 30 (33%) patients, 14 (15.4%) patients had detectable CTC with epithelial characteristics, 13 (14.3%) patients had CTC with EMT phenotype, and peripheral blood of 3 (3.3%) patients exhibit both CTC subtypes.

**Table 1:** Patient characteristics.

Variable	N	%
**All patients**	91	100.0
**T stage**		
T1	57	62.6
>T1	34	37.4
**Histology**		
IDC	75	82.4
Other	16	17.6
**Grade**		
Low/intermediate	48	52.7
High	41	45.1
Unknown	2	2.2
**N stage**		
N0	56	61.5
N+	34	37.4
Unknown	1	1.1
**Lymphovascular invasion**		
Present	23	25.3
Absent	68	74.7
**Hormone receptor status (cutoff 1%)**		
Positive for either	78	85.7
Negative for both	13	14.3
**HER2 status**		
Amplified	16	17.6
Negative	75	82.4
**p53 status**		
Present	32	35.2
Absent	58	63.7
**Bcl-2 status**		
Present	64	70.3
Absent	27	29.7
**Ki67**		
<14%	47	51.6
>14%	44	48.4
**Molecular subtype**		
Luminal A	42	46.2
Luminal B	21	23.1
HER2	16	17.6
Triple negative	12	13.2
**CTC status**		
CTC EP positive	14	15.4
CTC EMT positive	13	14.3
CTC EP and EMT positive	3	3.3
CTC ANY positive	30	33.0

CTC EP, circulating tumor cells with epithelial phenotype; CTC EMT, circulating tumor cells with epithelial-mesenchymal transition phenotype; CTC ANY, circulating tumor cells irrespective of phenotype; IDC, invasive ductal carcinoma.

### Association between plasma vitamin D level and patient/tumor characteristics

**Figure 1:**
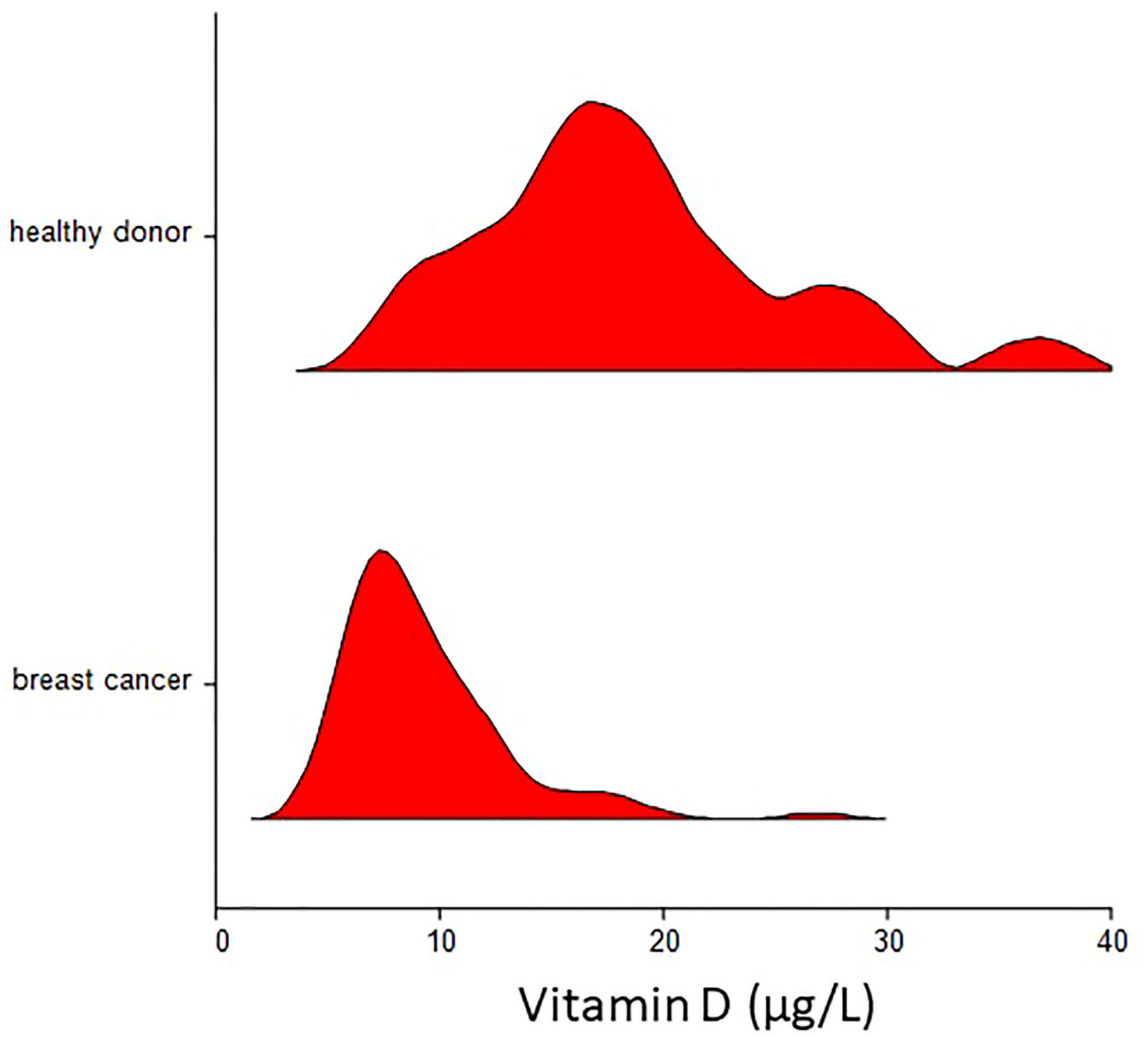
Violin plot of plasma vitamin D concentration in breast cancer patients and healthy donors. Mean ( ± SD) vitamin D plasma level was 9.3 ± 3.65 µg/L for breast cancer patients as compared to 18.6 ± 6.8 for healthy donors (p < 0.000001).

The characteristics of patients and the associations with plasma vitamin D levels are shown in **Table 2**. The mean ( ± SD) vitamin D plasma level was 9.3 ± 3.65 µg/L for breast cancer patients as compared to 18.6 ± 6.8 for healthy donors (p < 0.000001) (**Figure 1**). Regression analysis revealed that disease status (breast cancer patients vs. healthy donors) was independent of age associated with plasma vitamin D levels (p < 0.000001). There was no correlation between the month of the year when blood was drawn and vitamin D concentration (Spearman’s ρ = 0.14, p = 0.20).

**Table 2:** Association between plasma vitamin D level and patient/tumor characteristics.

Variable	N	Vitamin D plasma level (µg/L)		
All patients		Mean	SD	p-Value
**T stage**				
T1	57	9.17	3.14	0.90
>T1	34	9.50	4.41	
**Histology**				
IDC	75	9.26	3.71	0.72
Other	16	9.45	3.44	
**Grade**				
Low/intermediate	48	9.37	3.43	0.55
High	41	9.07	3.84	
**N stage**				
N0	56	9.39	3.00	0.26
N+	34	9.23	4.58	
**Lymphovascular invasion**				
Present	23	10.31	4.94	0.39
Absent	68	8.95	3.06	
**Hormone receptor status (cutoff 1%)**				
Positive for either	78	9.36	3.83	0.89
Negative for both	13	8.89	2.35	
**HER2 status**				
Amplified	16	11.20	6.09	0.38
Negative	75	8.89	2.77	
**p53 status**				
Present	32	10.23	4.62	0.13
Absent	58	8.73	2.90	
**Bcl-2 status**				
Present	64	9.55	4.02	0.57
Absent	27	8.69	2.53	
**Ki67**				
<14%	47	9.22	3.32	0.93
>14%	44	9.38	4.00	
**Molecular subtype**				
Luminal A	42	8.86	2.88	0.83
Luminal B	21	8.98	2.86	
HER2	16	11.20	6.09	
Triple negative	12	8.81	2.44	
**CTC status**				
CTC EP positive	17	9.08	4.90	0.21
CTC negative	62	9.62	3.50	
CTC EMT positive	16	8.16	1.99	0.12
CTC negative	61	9.69	3.49	
CTC ANY positive	30	8.50	3.89	**0.03**
CTC negative	61	9.69	3.49	

CTC EP, circulating tumor cells with epithelial phenotype; CTC EMT, circulating tumor cells with epithelial-mesenchymal transition phenotype; CTC ANY, circulating tumor cells irrespective of phenotype; IDC, invasive ductal carcinoma. Values of p ≤ 0.05 are considered as significant. Significant p values are in bold.

The plasma vitamin D level was not associated with any patient/tumor characteristics except CTC, where patients with detectable CTC in peripheral blood had significantly lower vitamin D levels as compared to patients without detectable CTC (mean ± SD 8.50 ± 3.89 vs. 9.69 ± 3.49 µg/L, p = 0.03). A similar trend was observed for both CTC subpopulations.

### Association between plasma vitamin D level, plasma calcium, urokinase plasminogen activator, plasminogen activator inhibitor-1, plasma cytokine and angiogenic factors, and MMP1 and MMP9 expressions in primary tumor

Plasma calcium level was available for 83 patients. There was no correlation between plasma vitamin D levels and calcium (Spearman’s rho = −0.0672, p = 0.55), including calcium levels corrected to albumin (Spearman’s rho = −0.1116, p = 0.32).

There was no association between D vitamin and TF, uPA, and/or PAI-1. Plasma vitamin D levels are inversely correlated with plasma TGF-β1, TGF-β2, IL β, IL-5, and eotaxin (all p < 0.05) (**Supplementary Table 1**). There was no association between D vitamin and expressions of MMP1 and MMP9 in primary tumor cells and tumor-associated stroma (**Supplementary Table 2** **).**

### Prognostic value of plasma vitamin D in primary breast cancer

**Figure 2:**
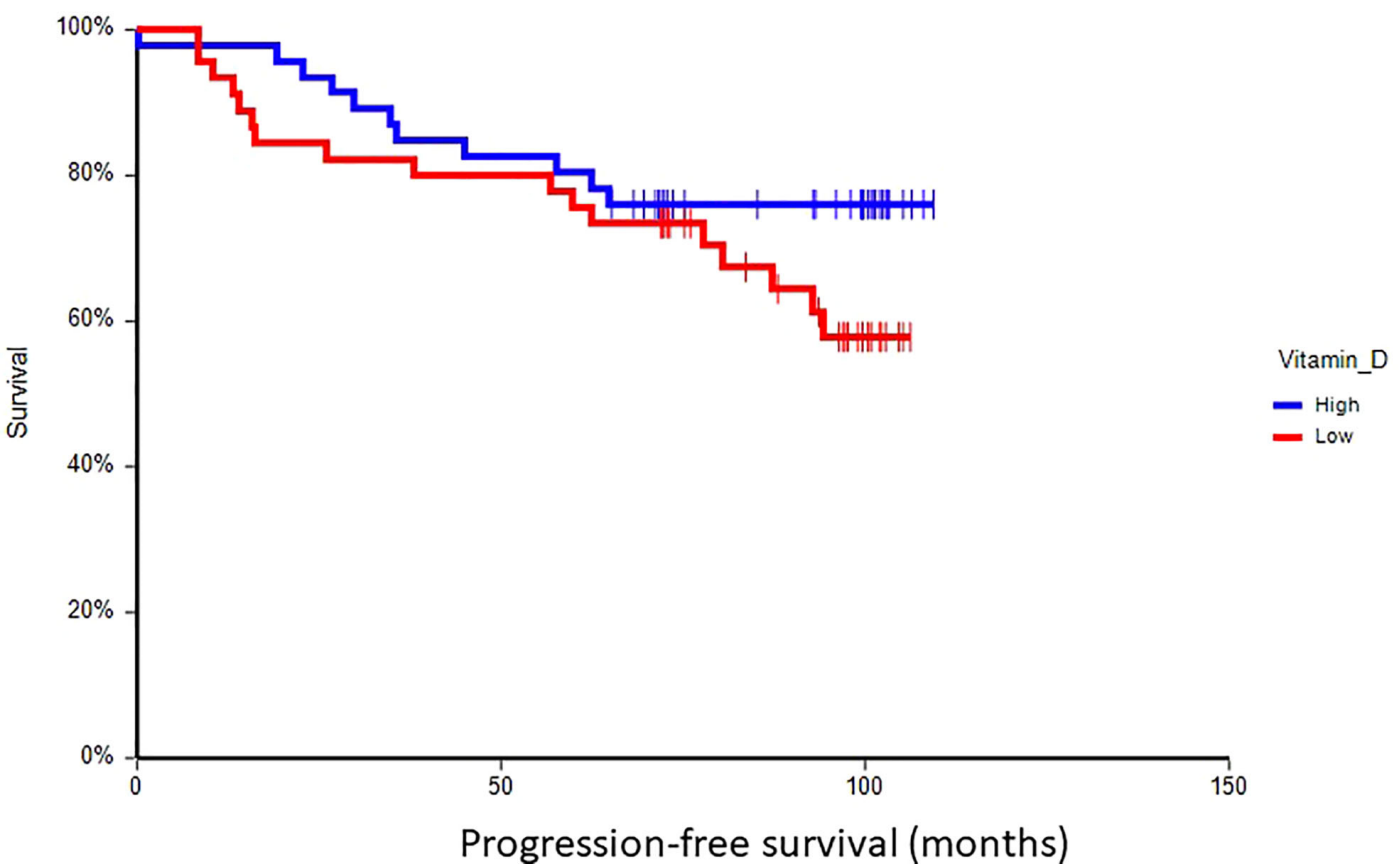
Kaplan–Meier estimates of probabilities of disease-free survival according to plasma vitamin D levels in primary breast cancer patients (N = 91). Patients with plasma vitamin D level above median had non-significantly better DFS as compared to patients with lower vitamin D level (HR = 0.59, 95% CI 0.28–1.24, p = 0.17; 0 = plasma vitamin D level below median, 1 = plasma vitamin D level above median). DFS, disease-free survival.

**Figure 3:**
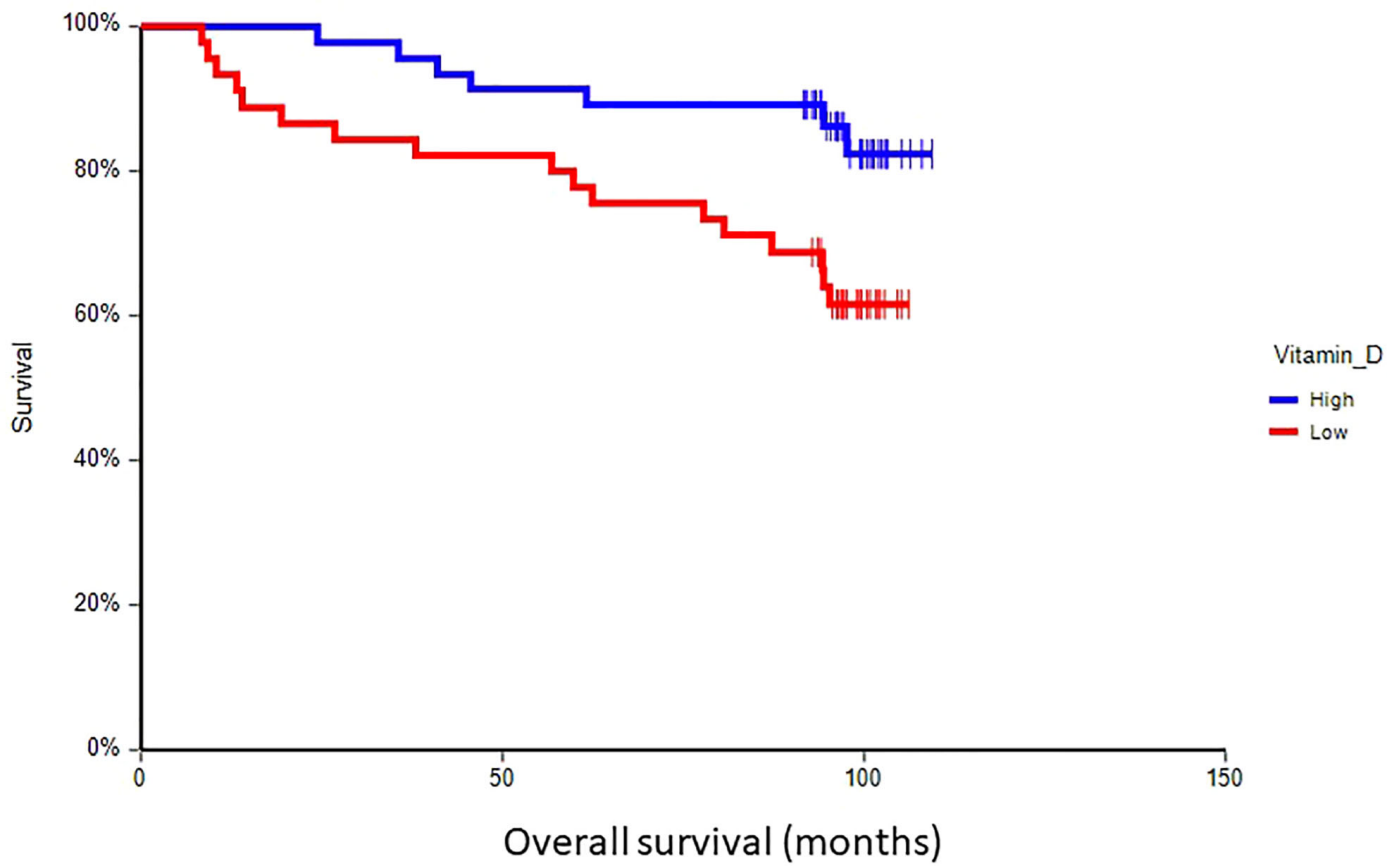
Kaplan–Meier estimates of probabilities of overall survival according to plasma vitamin D levels in primary breast cancer patients (n = 91). Patients with plasma vitamin D levels above median had significantly better OS as compared to patients with lower vitamin D levels (HR = 0.36, 95% CI 0.16–0.80, p = 0.017; 0 = plasma vitamin D level below median, 1 = plasma vitamin D level above median). OS, overall survival.

**Figure 4:**
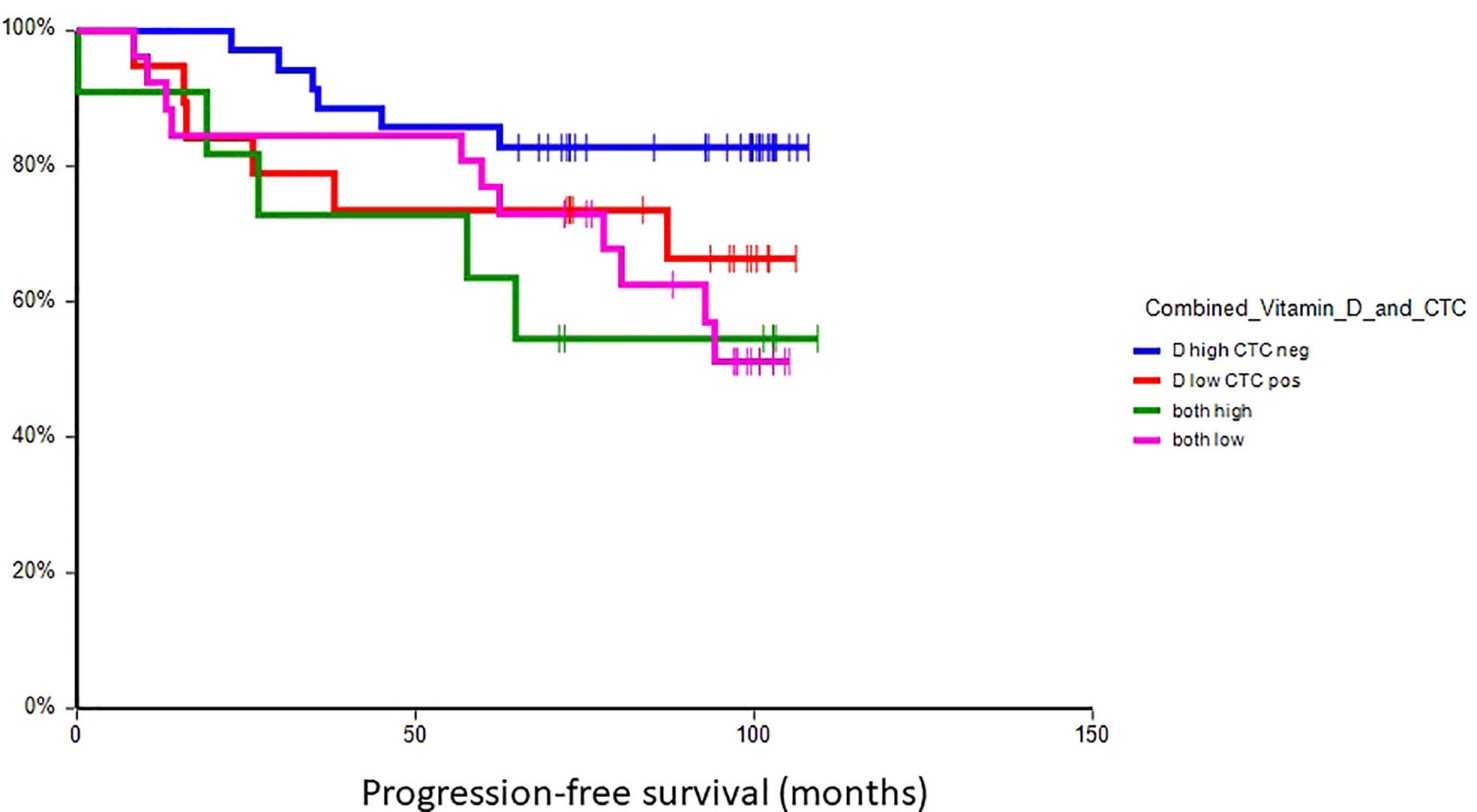
Kaplan–Meier estimates of probabilities of disease-free survival according to plasma vitamin D level and CTC status in primary breast cancer patients (N = 91). Patients with plasma vitamin D levels above median and undetectable CTC had significantly better DFS as compared to patients with lower vitamin D levels with CTC (HR = 0.48, 95% CI 0.14–1.60, p = 0.19). CTC, circulating tumor cell; DFS, disease-free survival.

**Figure 5:**
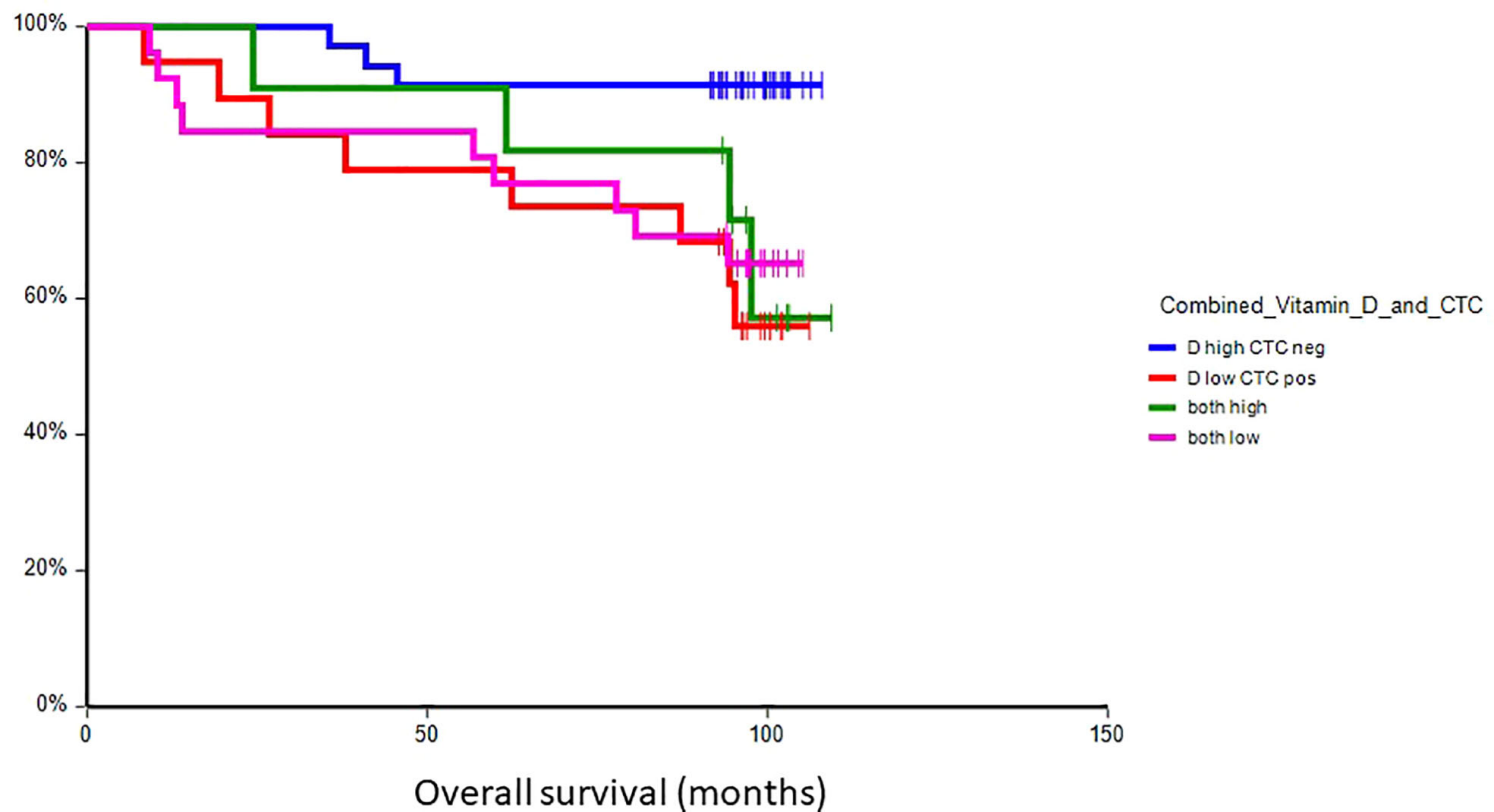
Kaplan–Meier estimates of probabilities of overall survival according to plasma vitamin D level and CTC status in primary breast cancer patients (n = 91). Patients with plasma vitamin D levels above median and undetectable CTC had significantly better OS as compared to patients with lower vitamin D levels with CTC (HR = 0.18, 95% CI 0.05–0.63, p = 0.004). CTC, circulating tumor cell; OS, overall survival.

At a median follow-up time of 96.5 months (range 8.4–109.4 months), 28 patients (30.8%) had experienced a DFS event, and 24 patients (26.4%) had died. Patients with vitamin D levels above median had better disease-free survival (hazard ratio (HR) = 0.59, 95% CI 0.28–1.24, p = 0.17) and overall survival (HR = 0.36, 95% CI 0.16–0.80, p = 0.017) as compared to patients with vitamin D levels below median (**Figures 2**, **3**). Combined prognostic values of CTC and vitamin D levels showed that the best prognosis was associated with CTC negativity and vitamin D levels above the median, while patients with detectable CTC and low vitamin D have the worst prognosis (HR = 0.48, 95% CI 0.14–1.60, p = 0.19 for DFS and HR = 0.18, 95% CI 0.05–0.63, p = 0.004 for OS) (**Figures 4**, **5**).

The prognostic value of plasma vitamin D levels was most pronounced in T1, invasive ductal cancer, HER2-negative disease, and high-grade disease, regardless of hormone receptor status, as well as in CTC-negative patients **(** **Table 3** **)**. Similarly, in luminal B and triple-negative subtypes, low plasma vitamin D levels were associated with inferior survival, while in luminal A and HER2-positive subtypes, plasma vitamin D was not prognostic.

**Table 3:** Prognostic value of vitamin D on disease-free survival and overall survival in primary breast cancer (vitamin D dichotomized below vs. above median).

	DFS				OS			
Variable	HR	95% CI low	95% CI high	p-Value	HR	95% CI low	95% CI high	p-Value
**All**	0.59	0.28	1.24	0.170	0.36	0.16	0.8	**0.017**
**T stage**								
T1	0.23	0.08	0.65	**0.013**	0.08	0.03	0.27	**0.002**
>T1	1.46	0.51	4.16	0.482	0.88	0.3	2.61	0.817
**Histology**								
IDC	0.56	0.26	1.21	0.143	0.31	0.13	0.72	**0.010**
Other	1.07	0.07	17.12	0.962	1.07	0.07	17.12	0.962
**Grade**								
Low and intermediate	0.55	0.16	1.91	0.349	0.42	0.08	2.07	0.296
High grade	0.68	0.27	1.71	0.421	0.37	0.15	0.93	**0.049**
**Lymph nodes**								
N0	0.5	0.12	1.99	0.327	0.28	0.07	1.11	0.092
N+	0.74	0.31	1.79	0.516	0.44	0.17	1.18	0.119
**Lymphovascular invasion**								
Absent	0.29	0.11	0.73	**0.020**	0.24	0.09	0.65	0.015
Present	1.39	0.39	5.01	0.629	0.52	0.14	1.99	0.324
**Hormone receptor status (cutoff 1%)**								
Negative for both	0.33	0.05	2.09	0.145	0.14	0.01	1.42	**0.009**
Positive for either	0.59	0.26	1.36	0.229	0.37	0.15	0.91	**0.047**
**HER2 status**								
Negative	0.49	0.2	1.17	0.115	0.26	0.1	0.68	**0.012**
Positive	0.94	0.23	3.74	0.924	0.71	0.16	3.12	0.650
**P53 status**								
Negative	0.53	0.23	1.26	0.167	0.35	0.14	0.87	**0.037**
Positive	0.96	0.21	4.32	0.961	0.49	0.08	2.87	0.421
**BCL-2**								
Negative	0.46	0.13	1.63	0.219	0.19	0.05	0.66	**0.005**
Positive	0.68	0.27	1.72	0.422	0.45	0.15	1.34	0.176
**Ki67 status (cutoff 14%)**								
<14%	1.09	0.27	4.35	0.907	0.49	0.1	2.44	0.402
>14%	0.43	0.18	1.03	0.060	0.28	0.11	0.72	**0.010**
**Molecular subtype**								
Luminal A	0.91	0.26	3.21	0.890	0.42	0.10	1.68	0.270
Luminal B	0.18	0.03	1.09	0.085	0.00	0.00	0.00	**0.021**
HER2	0.94	0.23	3.74	0.924	0.71	0.16	3.12	0.650
Triple negative	0.22	0.03	1.69	0.068	0.15	0.02	1.45	**0.012**
**CTC EP **								
Negative	0.45	0.2	0.98	**0.046**	0.22	0.09	0.56	**0.003**
Positive	3.36	0.32	34.83	0.293	1.38	0.27	6.95	0.694
**CTC EMT**								
Negative	0.48	0.19	1.18	0.114	0.34	0.13	0.89	**0.035**
Positive	1.42	0.36	5.52	0.601	0.52	0.12	2.29	0.415
**CTC ANY**								
Negative	0.36	0.14	0.95	**0.036**	0.22	0.07	0.71	**0.014**
Positive	1.52	0.44	5.24	0.483	0.78	0.24	2.48	0.679

CTC EP, circulating tumor cells with epithelial phenotype; CTC EMT, circulating tumor cells with epithelial-mesenchymal transition phenotype; CTC ANY, circulating tumor cells irrespective of phenotype; DFS, disease-free survival; OS, overall survival; IDC, invasive ductal carcinoma. Values of p ≤ 0.05 are considered as significant. Significant p values are in bold.

In a multivariate analysis, hormone receptor status, HER2 status, and lymph node involvement were independent predictors of disease-free survival, while vitamin D levels, hormone receptor status, and lymph node involvement were independent predictors of overall survival (**Table 4**).

**Table 4:** Multivariate analysis of factors associated with disease-free survival and overall survival.

DFS					OS			
Variable	Risk ratio	Lower 95.0% CL	Upper 95.0% CL	p-Value	Risk ratio	Lower 95.0% CL	Upper 95.0% CL	p-Value
**Vitamin D** High vs. low	0.56	0.24	1.30	0.1776	0.28	0.10	0.77	**0.0145**
**N stage** N+ vs. N0	5.43	2.35	12.55	**0.0001**	3.72	1.55	8.91	**0.0032**
**HR** HR+ vs. HR−	0.30	0.11	0.84	**0.0223**	0.29	0.09	0.93	**0.037**
**HER2** Amplified vs. negative	2.60	1.10	6.11	**0.0289**	2.20	0.88	5.47	0.0901

CTC EP, circulating tumor cells with epithelial phenotype; CTC EMT, circulating tumor cells with epithelial-mesenchymal transition phenotype; CTC ANY, circulating tumor cells irrespective of phenotype; DFS, disease-free survival; OS, overall survival. Values of p ≤ 0.05 are considered as significant. Significant p values are in bold.

## Discussion

In this translational study, we observed that patients with detectable CTCs in peripheral blood had significantly lower plasma vitamin D concentrations in comparison to patients without detectable CTCs. Mean plasma vitamin D levels for all study patients with lower as compared to normal plasma vitamin D levels in healthy individuals are consistent with previous observations of lower D vitamin levels in breast cancer patients (25, 37). Interestingly, there was no association between plasma vitamin D and other patient/tumor characteristics. We observed an association between vitamin D levels and plasma D-dimer, which is a marker of coagulation activation as well as some plasma cytokines. Moreover, patients with vitamin D above the median had better overall survival as compared to patients below median vitamin D, with the best outcome for patients with undetectable CTC and high plasma vitamin D levels. Subgroup analysis revealed that the prognostic value of vitamin D was most prominent in T1, invasive ductal cancer, HER2-negative disease, and high-grade disease, regardless of hormone receptor status, as well as in CTC-negative patients. In a previous study, an inverse association between vitamin D and prognosis was observed in luminal A and B subtypes, opposite to our study, where the prognostic value was observed in luminal B and triple-negative subtypes (38).

We suggest that the observed association between vitamin D and CTCs has a biological rationale. CTCs represent one of the key components of the metastatic cascade. Several factors that play important role in CTC intravasations including increased motility of cancer cells *via* EMT, degradation of the basal membrane by matrix metalloproteinases, and/or uPA system are affected by vitamin D (29, 30, 39). Our inverse correlation between vitamins and TGF-β1, TGF-β2, and IL-1β-supports this observation, as the TGF family are strong inducers of EMT (40, 41). Vitamin D attenuates the induction of EMT by TGF-β in colon carcinoma cells and inhibits *SNAIL1* and *SNAIL2* expressions and the E-cadherin/N-cadherin switch (42). Similarly, vitamin D attenuates IL-1β-induced EMT by inhibiting the expression of lncTCF7 (43). Vitamin D also could influence the survival of CTC in peripheral blood through its impact on other biological processes like cancer stem cell phenotype and/or immune system. Activation of the Wnt/b-catenin signaling pathway is accompanied by dedifferentiation, induction of EMT, and acquisition of stem cell properties (44). Vitamin D is a multilevel repressor of Wnt/b-catenin signaling in cancer cells (44). Moreover, macrophage-derived IL-1β-induced Wnt signaling is interrupted by vitamin D (45). We also observed an inverse relationship between vitamin D and IL-1β, IL-13, and eotaxin. While IL-1β is an inflammatory protein, IL-5 and eotaxin are both involved in allergic inflammation and asthma, but they are involved in breast cancer progression as well (46, 47). Vitamin D inhibits breast carcinoma cell migration, invasion, and metastatic capacities *via* a reduction of the expression/activity of several matrix metalloproteases (MMP1 and MMP9) and uPA/PAI and their inhibitors (29, 42, 48); however, in our study, there was no correlation between plasma vitamin D and plasma uPA/PAI, nor MMP1 and MMP9 expressions in the primary tumor.

Numerous studies showed an association between vitamin D levels and cancer outcomes, including breast cancer (49). However, interventional data that showed that vitamin D supplementation will have a positive impact on cancer incidence or prognosis are inconsistent (19, 50–54). One of the explanations is that vitamin D is a marker of biologically more aggressive disease, and a vitamin D decreased level is one of the characteristics of cancer disease analogically to decreased iron levels in chronic diseases with consequent anemia (55). Further studies are needed to dissect if vitamin D supplementation could decrease CTC and improve breast cancer outcomes.

There was no correlation between plasma vitamin D levels and calcium. The interaction between vitamin D and calcium is complex. The higher intake does not automatically lead to a higher plasma concentration. Vitamin D, parathormone, other dietary components, and many other factors do affect the absorption of calcium from the gut and also the metabolism of calcium in the bone. This explains why it is difficult to expect a strong significant correlation between vitamin D and calcium, more so in patients with breast cancer (56).

This study has several limitations, including small sample size and subsequent underrepresentation of some important subgroups like the HER2 molecular subtype. Another factor could be the time between blood draw and analysis of vitamin D, which was 10 years, which could be responsible for lower vitamin D levels for the entire group. Confounding factors could be related to sun exposure, which was not assessed, where patients with the worse condition could have less sun exposure and thus lower vitamin D; however, there was no correlation between the month of the year, when blood was drawn, and vitamin D concentration.

In conclusion, this is the first report of an association between plasma vitamin D concentrations and CTCs in primary breast cancer patients. Low vitamin D could be a consequence and hence a biomarker of a more invasive disease. Alternatively, vitamin D could be associated with survival because of its role in tumor dissemination. Whether its supplementation affects the metastatic cascade should be tested in animal experiments and interventional studies.

## Data availability statement

The original contributions presented in the study are included in the article/**Supplementary Material**. Further inquiries can be directed to the corresponding author.

## Ethics statement

This study was reviewed and approved by Ethical Committee of National Cancer Institute, Bratislava, Slovakia. The patients/participants provided their written informed consent to participate in this study.

## Author contributions

Conceptualization: PC and MM. Data curation: BV, GM, ZC, MK, JB, TS, KK, DP, and JM. Formal analysis: JM and MM. Funding acquisition: PC and MM. Investigation: BV, GM, ZC, MK, JB, TS, and KK. Methodology: BV, GM, ZC, TS, DC, PG, and KK. Project administration: PC, JM, and MM. Resources: JM, KK, PC, and MM. Validation: PC and MM. Visualization: MM. Writing—original draft: MM. Writing—review and editing: all authors. All authors contributed to the article and approved the submitted version.

## Funding

This research was funded by the Slovak Research and Development Agency (APVV), grant number APVV-16-0010, APVV-16-0178; by ERA-NET EuroNanoMed II INNOCENT; and by Scientific Grant Agency (VEGA), contracts No. 1/0724/11, 1/0044/15, 1/0271/17, and 2/0052/18.

## Acknowledgments

We would like to acknowledge Denisa Kolekova for her excellent technical help. We are grateful to all patients for their participation in the study.

## Conflict of interest

The authors declare that the research was conducted in the absence of any commercial or financial relationships that could be construed as a potential conflict of interest.

## Publisher’s note

All claims expressed in this article are solely those of the authors and do not necessarily represent those of their affiliated organizations, or those of the publisher, the editors and the reviewers. Any product that may be evaluated in this article, or claim that may be made by its manufacturer, is not guaranteed or endorsed by the publisher.
