# Supplementary material for: Vitamin D and circulating tumor cells in primary breast cancer
Source: Front Oncol. 2022 Sep 7;12:950451. doi: 10.3389/fonc.2022.950451 (PMC9489852; doi:10.3389/fonc.2022.950451)
Supplement: Supplementary file 1 [file Table_1.docx]

**Supplementary Table 1.** Plasma vitamin D level inversely correlated with plasma TGF-β1, TGF-β2, IL1b, IL5 and Eotaxin. Values of p ≤ 0.05 are considered as significant. Significant p values are in bold.

| **Variable** | **N** | **Mean** | **Median** | **SEM** | **SD** | **P-value** |
| --- | --- | --- | --- | --- | --- | --- |
| **TGF β1 (ng/mL)** |  |  |  |  |  |  |
| low vitamin D | 40 | 33540.3 | 21995.9 | 26250.2 | 3756.4 | **0.01515** |
| high vitamin D | 43 | 23864.0 | 15512.8 | 21182.4 | 3623.0 |  |
| **TGF β2 (ng/mL)** |  |  |  |  |  |  |
| low vitamin D | 39 | 2466.2 | 2321.2 | 676.0 | 111.3 | **0.00377** |
| high vitamin D | 43 | 2062.5 | 1861.0 | 711.9 | 106.0 |  |
| **IL-1β (ng/mL)** |  |  |  |  |  |  |
| low vitamin D | 40 | 6.2 | 4.8 | 4.7 | 0.6 | **0.04929** |
| high vitamin D | 44 | 4.4 | 3.5 | 3.4 | 0.6 |  |
| **IL-5** |  |  |  |  |  |  |
| low vitamin D | 40 | 11.5 | 9.4 | 8.3 | 1.2 | **0.04978** |
| high vitamin D | 44 | 8.7 | 6.4 | 6.5 | 1.1 |  |
| **Eotaxin (ng/mL)** |  |  |  |  |  |  |
| low vitamin D | 40 | 481.9 | 418.6 | 321.1 | 40.6 | **0.02725** |
| high vitamin D | 44 | 341.1 | 338.6 | 179.4 | 38.7 |  |
