# Supplementary material for: Vitamin D and circulating tumor cells in primary breast cancer
Source: Front Oncol. 2022 Sep 7;12:950451. doi: 10.3389/fonc.2022.950451 (PMC9489852; doi:10.3389/fonc.2022.950451)
Supplement: Supplementary file 2 [file Table_2.docx]

**Supplementary Table 2.** Correlation between vitamin D and MMP1 and MMP9 expression in tumor cells and tumor stroma

| **Variable** | **N** | **Mean** | **Median** | **SEM** | **SD** | **P-value** |
| --- | --- | --- | --- | --- | --- | --- |
| **MMP1 expression histoscore in tumor cells** |  |  |  |  |  |  |
| low vitamin D | 33 | 54.5 | 25.0 | 68.7 | 11.8 | 0.3315 |
| high vitamin D | 43 | 40.0 | 10.0 | 67.0 | 10.3 |  |
| **MMP1 expression histoscore in tumor associated stroma** |  |  |  |  |  |  |
| low vitamin D | 32 | 52.5 | 20.0 | 70.4 | 12.1 | 0.2702 |
| high vitamin D | 43 | 42.4 | 10.0 | 67.2 | 10.5 |  |
| **MMP9 expression histoscore in tumor cells** |  |  |  |  |  |  |
| low vitamin D | 37 | 149.9 | 170.0 | 93.3 | 13.9 | 0.8878 |
| high vitamin D | 41 | 150.4 | 150.0 | 76.4 | 13.2 |  |
| **MMP9 expression histoscore in tumor associated stroma** |  |  |  |  |  |  |
| low vitamin D | 36 | 81.5 | 70.0 | 68.5 | 10.3 | 0.3889 |
| high vitamin D | 41 | 64.9 | 50.0 | 55.1 | 9.6 |  |
